# *Sca*1, a previously undescribed paralog from autotransporter protein-encoding genes in *Rickettsia *species

**DOI:** 10.1186/1471-2180-6-12

**Published:** 2006-02-20

**Authors:** Maxime Ngwamidiba, Guillaume Blanc, Didier Raoult, Pierre-Edouard Fournier

**Affiliations:** 1 Unité des rickettsies, IFR 48, CNRS UMR 6020, Faculté de médecine, Université de la Méditerranée, 27 Boulevard Jean Moulin, 13385 Marseille cedex 05, France; 2 Information Génomique et Structurale, UPR 2589, 31, Chemin Joseph Aiguier, 13402 Marseille Cedex 20, France

## Abstract

**Background:**

Among the 17 genes encoding autotransporter proteins of the "surface cell antigen" (*sca*) family in the currently sequenced *Rickettsia *genomes, *ompA*, *sca*5 (*ompB*) and *sca*4 (gene D), have been extensively used for identification and phylogenetic purposes for *Rickettsia *species. However, none of these genes is present in all 20 currently validated *Rickettsia *species. Of the remaining 14 *sca *genes, *sca*1 is the only gene to be present in all nine sequenced *Rickettsia *genomes. To estimate whether the *sca*1 gene is present in all *Rickettsia *species and its usefulness as an identification and phylogenetic tool, we searched for *sca*1genes in the four published *Rickettsia *genomes and amplified and sequenced this gene in the remaining 16 validated *Rickettsia *species.

**Results:**

*Sca*1 is the only one of the 17 rickettsial *sca *genes present in all 20 *Rickettsia *species. *R. prowazekii *and *R. canadensis *exhibit a split *sca*1 gene whereas the remaining species have a complete gene. Within the *sca*1 gene, we identified a 488-bp variable sequence fragment that can be amplified using a pair of conserved primers. Sequences of this fragment are specific for each *Rickettsia *species. The phylogenetic organization of *Rickettsia *species inferred from the comparison of *sca*1 sequences strengthens the classification based on the housekeeping gene *gltA *and is similar to those obtained from the analyses of *ompA, sca*5 and *sca4*, thus suggesting similar evolutionary constraints. We also observed that Sca1 protein sequences have evolved under a dual selection pressure: with the exception of typhus group rickettsiae, the amino-terminal part of the protein that encompasses the predicted passenger domain, has evolved under positive selection in rickettsiae. This suggests that the Sca1 protein interacts with the host. In contrast, the C-terminal portion containing the autotransporter domain has evolved under purifying selection. In addition, *sca*1 is transcribed in *R. conorii*, and might therefore be functional in this species.

**Conclusion:**

The *sca*1 gene, encoding an autotransporter protein that evolves under dual evolution pressure, is the only *sca-*family gene to be conserved by all *Rickettsia *species. As such, it is a valuable identification target for these bacteria, especially because rickettsial isolates can be identified by amplification and sequencing of a discriminatory gene fragment using a single primer pair. It may also be used as a phylogenetic tool. However, its current functional status remains to be determined although it was found expressed in *R. conorii*.

## Background

Members of the genus *Rickettsia *are obligate intracellular, Gram-negative, bacteria. These bacteria are surrounded by a crystalline proteic layer [1], also referred to as S-layer, which represents 10 to 15 % of their total protein mass [2] and is made of immunodominant surface protein antigens (SPA) [3-5]. Prior to *Rickettsia *genome sequencing, two SPAs, i.e. rOmpA [6,7] and rOmpB [8,9], were identified in *Rickettsia *species. These two high molecular weight proteins are major antigenic determinants eliciting an early and dominant immune response in patients infected with rickettsiae [10]. Recent studies suggested that these two proteins are involved in adhesion to host cells [11,12]. This assumption is supported by the presence of conserved repeated peptide motifs which are common in some adhesive proteins from other species [13].

rOmpA and rOmpB are encoded by the *omp*A [14] and *sca*5 (or *omp*B) [15] genes, respectively. Sequence analyses readily identify the presence of a highly conserved autotransporter β-barrel domain (hereafter designated as "autotransporter domain") at the C-termini of the protein products. Proteins carrying this domain are collectively designated as "autotransporter proteins", and have been described in many Gram-negative bacteria [16]. The autotransporter domain forms a β-barrel pore in the bacterial outer membrane, and allows the amino-terminal part ("passenger domain") of the precursor to be exported across the outer membrane of the bacterium, and later be released following cleavage of the autotransporter domain. Theoretical molecular masses of the predicted proteins derived from *omp*A and *omp*B genes exceed those experimentally measured for rOmpA and rOmpB, respectively [3,4,9,17], implying the cleavage of the autotransporter domain from their precursors. Indeed, such a post-translational processing was demonstrated for rOmpB for which a ≈30-kDa peptide is cleaved from the C-terminus of the protein [3,4,9,17,18]. Another gene, annotated as *sca*4 (surface cell antigen 4), previously named gene D [19] and known to encode a 120-kDa intracytoplasmic protein, has a passenger domain similar to those of *ompA *and *ompB*. However, it lacks the autotransporter domain [18].

Based on the sequences of nine *Rickettsia *genomes, we have recently identified another 14 autotransporter genes exhibiting structures similar to those of *ompA *and *ompB *[18]. Members of the paralogous *sca *gene family are diversely degraded and distributed among *Rickettsia *species, with numbers of complete genes ranging from two in *R. prowazekii *to 10 in *R. felis *[18]. Among the 17 *sca *genes, only three are present in all nine *Rickettsia *genomes, *i.e., sca*1, *sca*4, and *sca*5 (*ompB*). However, we have previously demonstrated that *sca*5 was not amplified in *R. canadensis *[15] and that *sca*4 was fragmented in *R. prowazekii *and not amplifiable in *R. canadensis *[19]. As a consequence, none of the previously studied *sca *genes, despite their demonstrated usefulness for identification of these bacteria, may serve to identify all rickettsial isolates. Likewise, no current phylogenetic study based on *sca *genes included all *Rickettsia *species.

Of the 17 rickettsial autotransporter proteins putatively encoded by the 17 *sca *genes, only rOmpA and rOmpB have been detected by SDS-PAGE or western-blotting in *R. conorii *[10] as well as Sca4 in *R. japonica *[20]. Curiously, neither *omp*A nor *omp*B are conserved in all validated *Rickettsia *species, despite their seemingly important roles in adhesion to host cells [11]. No protein product has been identified yet for the other *sca *genes, raising the question as to whether those genes are functional. By RT-PCR, we recently observed that *sca*2 was transcribed in *R. conorii *[21]. In this study, to further characterize the *sca *family of genes, we studied the *sca*1 gene, the only gene together with *ompB *and *sca*4 present in the nine *Rickettsia *genomes [18]. By comparing *sca*1 gene sequences from all 20 validated *Rickettsia *species, we performed a phylogenetic analysis of *Rickettsia *species and analyzed more precisely the evolutionary forces that acted on *sca*1. In addition, as *ompB *and *sca*4 are expressed, we also examined the transcriptional status of the *sca*1 gene in *R. conorii*.

## Results

### *Sca*1 sequences

Orthologous genes of the *R. conorii sca*1 gene in the genomes of *R. prowazekii*, *R. typhi*, and *R. felis*, were readily identified with the use of BLASTn, tBLASTn, and BLASTp homology searches. In *R. prowazekii*, the *sca*1 gene was split into three consecutive ORFs (RP016 to RP018).

*Sca*1 fragments were amplified from all tested *Rickettsia *species. All negative controls remained negative. The size of *sca*1 genes ranged from 1,782 bp for *R. bellii *to 5,928 bp for *R. japonica. *All studied species exhibited distinct *sca*1 sequences, which were deposited in GenBank under the accession numbers reported in Table 1. The global pairwise nucleotide sequence identities of *sca*1 varied from 57% between *R. prowazekii *and *R. canadensis *to 99 % between *R. sibirica *and *R. parkeri*. This variation is greater than that observed for the 16 S rDNA (> 97.2%) and *gltA *genes (> 85 %), previously studied for all *Rickettsia *species [22,23]. This variation is also greater than that observed for *sca*5, which exhibits pairwise identities ranging from 70 to 99.6 % [15]. Thus *sca*1 appeared to be more divergent at the nucleotide level among *Rickettsia *species than *sca*5. The G+C% of *sca*1 sequences ranged from 30.15 % for *R. prowazekii *to 34.68 % for *R. helvetica*. Differences among *Rickettsia *species consisted of nucleotide substitutions, insertions and deletions, but not of variations in number of repeats as observed in *ompA *[24]. Insertions and/or deletions were found in all studied species and varied in size and position according to the species. When translated into amino acids, *sca*1 sequences were free from internal stop codons except *R. prowazekii *and *R. canadensis*. The overall pairwise amino acid sequence identities ranged from 37 % between *R. canadensis *and *R. bellii *to 97% between *R. conorii *and *R. sibirica*.

Using the SVARAP software, we identified a 488-bp fragment at the 3'-end of the *sca*1 gene, in the autotransporter domain. With regard to the *R. japonica sca*1 sequence, this fragment was located between nucleotides 4,894 and 5,930. It was flanked by conserved sequences within which it was possible to identify the primer pair F1MAX: 5'-AAGAGGTYTRTGGATGCGT-3' and RMAX: 5'-GAYAATATATTATTYTCTTTC-3'. The specificity of these primers was confirmed by comparison with sequences available in the GenBank database. Each of the 20 *Rickettsia *species we studied had a specific fragment sequence, with pairwise nucleotide sequence identities varying from 78.5% between *R. belli *and *R. prowazekii *to 99.8 % between *R. parkeri *and *R. africae*. Fragment sequences were deposited in GenBank under the accession numbers reported in Table 1.

### Phylogenetic analysis

**Figure 1:**
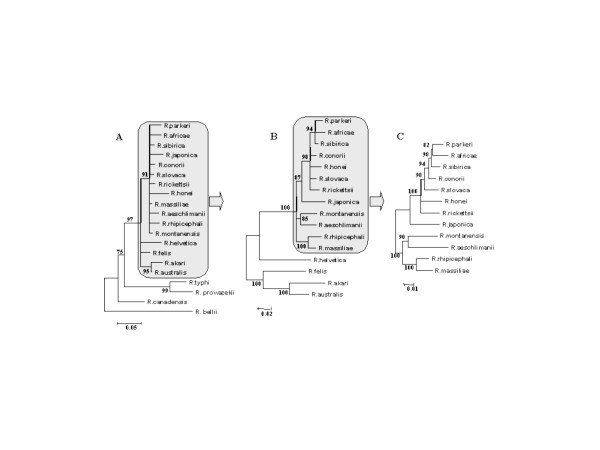
Dendrogram representing phylogenetic relationships between *Rickettsia *species inferred from comparison of Sca1 amino acid sequences. The Neighbor-Joining phylogenetic tree was inferred with the MEGA software version 2.1. The numbers at the nodes are the proportion of 100 bootstrap resamplings that support the topology shown. The scale bar indicates a 5 % sequence divergence. 1A: phylogeny of all 20 species using an alignment of the autotransporter domain; 1B: phylogeny of spotted fever group rickettsiae inferred from the comparison of full Sca1 sequences; 1C: phylogenetic resolution of members of the *R. rickettsii *and *R. massiliae *clusters.

The phylogenetic relationships among *Rickettsia *species were inferred in three steps. Because the passenger domains of the Sca1 proteins could not be aligned reliably owing to a high level of divergence between distant *Rickettsia *species, we first reconstructed the phylogeny of the 20 species from the alignment of the autotransporter domains, which is much more conserved. The autotransporter domain alignment contained 282 amino acid sites. In this phylogenetic tree (Figure 1A), the three major recognized *Rickettsia *clades (spotted fever, typhus and *R. bellii *groups) were well separated with bootstrap value >75%. In addition, R. *canadensis *appeared to form a separate fourth deep branching clade. The spotted fever sub-tree was poorly resolved, with most branches having bootstrap values <75%. We realigned separately the Sca1 proteins from spotted fever group (SFG) rickettsiae, including the passenger domain, and used them for phylogenetic reconstruction. After removing gaps and ambiguous positions, the SFG alignment contained 628 amino acid sites and 16 sequences. The SFG tree (Figure 1B) was organized in three distinct clades. The first clade included *R. felis*, *R akari*, and *R. australis*; the second clade contained a single species, *i.e., R. helvetica*. The last clade contained all other SFG species, including *R. conorii*. This latter clade, poorly resolved, was therefore re-analysed separately. The alignment of the Sca1 proteins from the latter clade contained 12 sequences and 1,211 amino acid sites after gap removal. The resulting phylogenetic tree was entirely resolved with all branches supported with bootstrap values > 82%, except for the placement of *R. honei *with a boostrap support of 53% (Figure 1C). Two clusters supported by bootstrap values of 100% were observed, one including *R. massiliae, R. rhipicephali, R. aeschlimannii *and *R. montanensis*, and the other including all remaining species.

### Study of evolutionary forces

**Figure 2:**
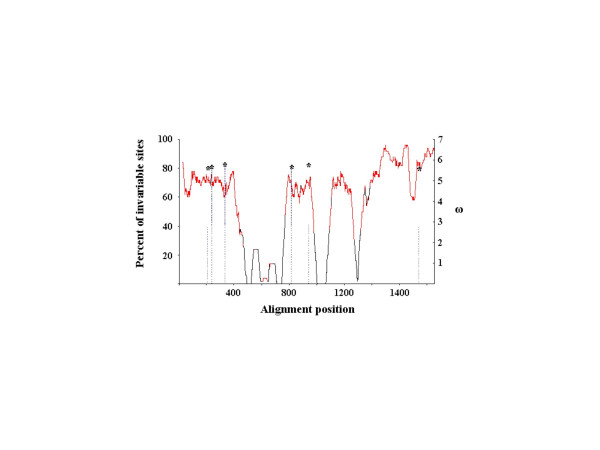
Variable selective pressure along the Sca1 sequences. The x axis represents the alignments of the Sca1 sequences. The predicted passenger and autotransporter domains expand from positions 1 to 800, and from positions 801 to the end, respectively. The N-terminal signal peptide is not included in the alignment because the sequence was not determined for some of the *Rickettsia *species under study. The y axis represents the percent of invariable sites per window of 50 sites attributed to each amino acid position in the Sca1 protein alignment. Predicted positively selected sites and their mean of ϖ (calculated as the average of ϖ over the 11 site classes of model M8, weighted by the posterior probabilities; right axis) appear as stars. The curve represents the averages of means of ϖ calculated for sliding windows of 50 codon sites (right axis).

We carried out a sliding window analysis to highlight the local variations of sequence similarity along the protein sequences. Two broad regions with different levels of sequence divergence could be distinguished (Figure 2). The first region, which encompasses the majority of the alignment (from position 1 to 800), is characterized by a higher level of sequence divergence with 50 aa window not exceeding 86% of invariable positions and dropping at values as low as 60% at positions 127–186 and 325–385 (Figure 2). The second region from position 801 to the end, overlaps with the autotransporter domain and is more conserved with 50 aa window ranging from 78% to 98% of invariable positions. This heterogeneity of sequence divergence suggests different strength of selective constraints in the different domains of the Sca1 protein.

To further characterize the variations of selective constraints along the Sca1 protein sequence, models of codon substitutions were fitted to the aligned Sca1 codon sequences to estimate the ω ratios (Table 3). Likelihood ratio tests were applied between nested models to select the one that best described the data (Table 4). Models M1, M2 and M3, which account for variable ω ratio among codon sites are all significantly better (P < 0.0001) than model M0, which accounts for a single ω ratio for all codon sites in the alignment. This confirms that selective constraints are not homogenous along the Sca1 sequences. In addition, model M2 (selection) significantly improved the likelihood over model M1 (neutral). Interestingly, models M2 and M3 both predicted a class of codon sites with ω > 1, suggesting that some amino acid positions are under positive selection. This result was further confirmed by the model M8, which better fit the data than model M7 and predicted an additional class of codons with ω = 5.75. Note that model M2, M3 and M8 identified the same amino acid positions under positive selection. Thus, the signal for positive selection was consistent whatever the evolutionary model considered (Table 3). The predicted positively selected sites were mainly distributed within the internal passenger domain (Figure 2) except site 1049, which fall in the AT domain. Unfortunately, little is known about the Sca1 passenger domain and no information is available on the 3-dimensional proximity of the positively selected sites. The C-terminal region encompassing the autotransporter domain was globally under stronger purifying selection (Figure 2). Models M3 and M8 predicted that 44% and 45% of codon sites respectively (mostly located in the N-terminal region) belong to codon categories with ω close to or higher than 1. This suggests that a substantial fraction of amino acid sites in the passenger domain of Sca1 proteins was under weak or positive selection. When estimating the Ka/Ks ratios by branch, we observed that the ratios were > 1 among SFG rickettsiae but < 1 between *R. prowazekii *and *R. typhi*, thus suggesting a different evolutionary pressure that acted on Sca1 in SFG and typhus group rickettsiae.

### RT-PCR assay

An RT-PCR product of 407-bp was obtained from *R. conorii*. Negative controls remained negative. The sequenced RT-PCR product was 100% identical to the *sca1 *sequence from *R. conorii*.

## Discussion

**Figure 3:**
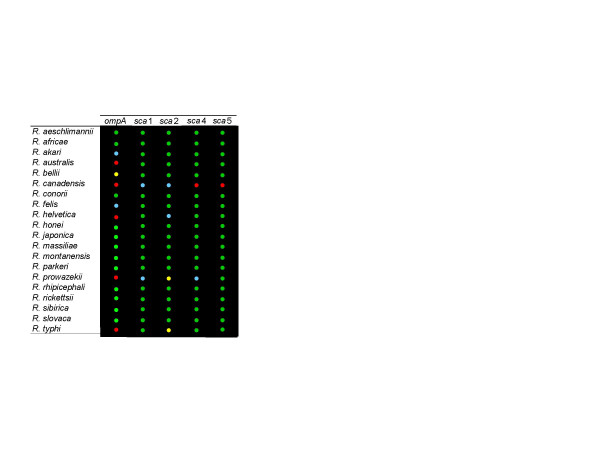
Current knowledge on the distribution of the *ompA*, *sca*1, *sca*2, *sca*4, and *sca*5 genes among the 20 validated *Rickettsia *species. Green dots indicate complete genes, whereas blue dots indicate split genes, yellow dots indicate gene fragments, and red dots indicate absent genes.

We studied the *sca*1 gene in the 20 currently validated *Rickettsia *species and demonstrated that this gene, the only *sca *gene present in all 20 species (Figure 3), is a useful tool for the identification and phylogenetic study of these bacteria.

We obtained unique *sca*1 sequences for each of the 20 studied species. We also observed that *sca*1 sequences exhibit a greater level of interspecies variablity than previously studied *sca *genes, thus making *sca*1 a potential identification tool for rickettsiae.*Sca*1 loci exhibit diverse levels of conservation among *Rickettsia *species. It is present as a complete gene in all studied species except in *R. prowazekii *and *R. canadensis*, in which it is present as a pseudogene. Amiri *et al. *have previously demonstrated that the rate of gene degradation may vary among *Rickettsia *species, with typhus group rickettsiae exhibiting a higher rate than spotted fever group rickettsiae [25]. In our study, we observed a higher degradation gradient in typhus group (*R. prowazekii *but not *R. typhi*) than in spotted fever group rickettsiae (none of 18 species studied). We have previously observed such variations for *ompA*, *sca*2, *sca*5, and *sca*4 (Figure 3). *ompA *is present as a pseudogene in *R. felis *and *R. akari*, as fragments in *R. bellii *[18], and could not be amplified in *R. helvetica*, *R. canadensis*, and typhus group rickettsiae [14]; *sca*2 is present as a pseudogene in *R. helvetica *and *R. canadensis*, and as remnants in typhus group rickettsiae [21]; *sca*4 was split in *R. prowazekii *and could not be detected in *R. canadensis *[19]; and *sca*5 was not found in *R. canadensis *[15]. All other *sca *genes are even less conserved among *Rickettsia *species [18]. Thus, *sca*1 is the only *sca *family gene to be present in all 20 validated *Rickettsia *species. This specificity and the length of the gene prompted us to search for a gene fragment that could serve as an identification tool for all species. We identified a 488-bp fragment within the autotransporter domain whose sequence could distinguish all *Rickettsia *species. This sequence fragment has the advantage of being amplified using a single primer pair (F1MAX-RMAX), and thus may be a useful tool for both detection and identification of *Rickettsia *species.

Due to high levels of divergence between distant *Rickettsia *species in their passenger domains, we based our phylogenetic analysis on a polyphasic approach. The *sca*1-based phylogenetic analysis of *Rickettsia *species was well supported for most species (Figure 1). Four clusters were identified and supported by elevated bootstrap values: one included the *Rickettsia *species previously classified within the *R. rickettsii *group [26]; a second was made of members of the *R*. *massiliae *group; a third incorporated members of the *R. akari *group; and the fourth group was made of members of the typhus group. By comparison with previous phylogenetic studies based on autotransporter genes or *gltA*, involved in a central metabolic pathway [14,15,22], we obtained similar organizations among SFG rickettsiae. Obtaining similar phylogenetic reconstructions from the analyses of different genes with different functions suggests that the true phylogenetic organization of members of the *Rickettsia *genus is close to that obtained in our study.

By comparing *sca *genes among nine *Rickettsia *genomes, we have previously observed that five of the 17 *sca *genes (*ompA, sca*1, *sca*2, *sca*4 and *sca*5) have evolved under positive selection [18]. However, this analysis was global and based on a limited number of species. Herein, we conducted a detailed study using a sliding-window approach. This method showed that *sca*1 is divided into two domains undergoing different selection pressure (Figure 2). The 3'-end of the gene, highly conserved among species and encoding the autotransporter domain, was found to evolve under purifying selection. This is likely due to the complex structure of the β barrel pore, which involves numerous specific interactions between amino acids. Amino acid replacements in the autotransporter domain may be more likely to be deleterious for the protein function. In contrast, the 5'-part of the gene, highly variable among species and encoding the secreted part of the protein, has evolved under weaker or neutral selection. In addition, we have shown here that some amino acid positions within the N-terminal domain have evolved under positive selection. The immune system of the host is known to drive positive Darwinian selection for diversity of immuno-exposed proteins such as the *por*B gene of *Neisseria gonorrhoeae *[27]. To date, only the rOmpA, rOmpB, and Sca4 proteins have evidence of expression and are known to induce an immune response in humans [14,15,20]. The functional status of the other *sca *genes is still uncertain. Several features of *sca*1 evolution suggest that this gene is functional in most of the *Rickettsia *species belonging to the spotted fever group. First, if the process of degradation started before the separation of these species, we would expect the level of divergence to be homogeneous along the protein sequences. The fact that we could distinguish two regions with different levels of sequence divergence argues against the pseudogene hypothesis. In addition, the genic region corresponding to the autotransporter domain appeared to evolve mainly under purifying selection, which is characteristic of functional coding sequences. In addition, we observed that *sca*1 has evolved under distinct evolutionary patterns depending on the phylogenetic branches. In typhus group rickettsiae, Sca1 has evolved under purifying selection whereas in other species, it has evolved under positive selection. Whether this is linked to a difference in vector or host is unknown.

Although *sca*1, *sca*4 and *sca*5 are the most conserved *sca *genes among *Rickettsia *species (Figure 3), only the latter two genes are known to be expressed. Thus, we investigated the transcription of this gene, which is currently unknown. Chao *et al. *could not find *sca*1 in the *R. prowazekii *proteome [28]. However, this may be explained by the fact that *sca*1 is a pseudogene in this species. Using RT-PCR, we demonstrated that *sca*1 was transcribed at least in *R. conorii*. The molecular mass of the predicted mature Sca1 protein in *R. conorii *being 170 kDa, which is close to that of rOmpA, it is possible that the expression of Sca1 has not been detected previously because of the lack of resolution of SDS-PAGE analyses. Future efforts will be put to determine whether the transcription of *sca*1 is followed by its translation into a protein.

## Conclusion

We have demonstrated that the *sca*1 gene, which encodes a putative autotransporter protein, is the only *sca *gene present in all *Rickettsia *species. We identified a 488-bp variable *sca*1 fragment amplifiable using a pair of conserved primers that may be used as a detection and identification tool for *Rickettsia *species. Phylogenetic relationships obtained from the analysis of this gene are consistent with those obtained from the analyses of other genes, thus suggesting that this gene is undergoing similar evolutionary constraints. The *sca*1 gene, which is partially degraded in certain species, is undergoing a dual selection pressure and is transcribed into mRNA in *R. conorii*, thus suggesting that it might be functional. However, as its putative protein product has not been detected as yet, further studies should aim at determining whether it plays a role in the immune response observed in patients with rickettsioses.

## Methods

### Rickettsial strains

The strains used in this study are listed in Table 1. All rickettsiae were grown on Vero cell monolayers as previously described [22]. When Gimenez staining [29] showed the cells to be heavily infected, they were harvested and centrifuged at 1,200 g for 10 min, resuspended in MEM (minimum essential medium, GIBCO) and stored at -70°C until DNA extraction was performed.

### PCR amplification and DNA sequencing

*Sca*1 gene sequences were retrieved from the four published *Rickettsia *genomes, *i.e.*, *R. conorii *(NC_003103), *R. prowazekii *(NC_000963), *R. typhi *(NC_006142), and *R. felis *(NC_007109). Primers used for amplification and sequencing are presented in Table 2. They were designed by aligning the *sca*1 sequences from *R. conorii *and *R. felis*.

For PCR, genomic DNA was extracted using the Chelex method as previously described [30]. PCRs were carried out in a PTC-200 automated thermal cycler (MJ Research, Waltham, MA) using the eLONGase DNA polymerase (Gibco-BRL, Gaithersburg, USA). The 25 μl reaction mixture consisted of (final concentration): primers 12.5 pmol each, MgCl_2 _40 mM, dNTP 5 mM each, 2.5 μl of buffer 10×, 0.5 μl eLONGase DNA polymerase, 5 μl of DNA, and sterile water to a final volume of 25 μl. PCR amplification was performed under the following conditions: a 3-min denaturation step at 94°C followed by 40 cycles of denaturation for 30 s at 94°C, annealing for 30 s at 50°C and extension for 90 s at 68°C. Holding for 7 min at 68°C to allow complete extension of the PCR products completed the amplification. PCR products were separated by electrophoresis on 1% agarose gels, visualized by staining with ethidium bromide and then purified using the QIAquick PCR purification kit (QIAGEN, Hilden, Germany) as described by the manufacturer. PCR products were sequenced twice in both directions using the d-Rhodamine Terminator Cycle Sequencing Ready Reaction kit (PE Applied Biosystems, Warrington, UK) as proposed by the manufacturer. Sequencing primers were the same as PCR primers. Sequencing products were resolved using an ABI 3100 automated sequencer (PE Applied Biosystems) following the manufacturer's instructions. For each species the sequence of both DNA strands was determined twice.

When primers failed to amplify a gene fragment, we used GenomeWalker DNA walking method. DNA was extracted using the FastDNA kit and the FastPrep instrument (Bio101, Carlsbad, CA) according to the manufacturer's instructions. Sequencing was performed using the Universal Genome Walker Kit (CLONTECH, Palo Alto, CA) as proposed by the manufacturer. When GenomeWalker failed to provide *sca*1 sequences, a mini shotgun assay was performed using the Eppendorf Perfectprep Plasmid 96 Vac Direct Bind kit (Brinkmann, Westbury, NY) as described by the manufacturer.

Sequence assembly was performed using the software package ABI Prism DNA sequencing analysis software version 3.0 (PE-Applied Biosystems). Sequences were deposited in GenBank under the accession numbers detailed in Table 1.

### Identification of a molecular identification target

To identify *sca*1 sequence fragments variable among *Rickettsia *species and useable as a PCR target, we used the Sequence Variability Analysis Program (SVARAP) software [31]. This software determines the genetic diversity among sequences using a sliding window analysis, by moving a window of 50 nucleotide positions along the alignment with a step of one. We focused on fragments of a maximum size of 500-bp flanked by conserved sequences in which selection of primers was possible.

### Phylogenetic reconstruction

*Sca*1 nucleic and amino acid sequences were aligned using the CLUSTALW software [32]. Neighbor-Joining (NJ) and Maximum Parsimony (MP) phylogenetic reconstructions were carried out using the MEGA2 software (version 2.1) [33]. Maximum Likelihood (ML) trees were performed using the programs PROTML (MOLPHY software package) [34] and PAUP [35] for the protein and nucleotide alignments, respectively. For the NJ and ML analyses, we used the JTT model of substitutions for proteins and the TN93 model for nucleotides. Statistical support for internal branches was assessed by bootstrap sampling for MP and NJ analyses and Resampling of Estimated Log-Likelihood (RELL) for ML analyses [36].

### Analysis of evolutionary forces acting on *sca*1

The G+C content statistics were obtained using FREQSQ on the Infobiogen website [37].

Alignments were manually corrected to remove gapped and ambiguous positions. The sliding-window analysis was carried out by moving a window of 50 amino acid positions along the alignment with a step of one. For each window, we recorded the percentage of positions with identical residues in all sequences. The passenger domains of the Sca1 proteins were difficult to align between distant *Rickettsia *owing to a high level of sequence divergence. We therefore restricted our analysis to the 12 *Rickettsia *species presented in Figure 1C to obtain a reliable multiple alignment.

To study the selective forces acting on *sca*1 proteins, we determined the ratio ω = Ka/Ks where Ka and Ks are the rates of non-synonymous and synonymous substitution accumulations, respectively. In absence of codon utilization bias, as is the case for *Rickettsia *genes [38], synonymous substitutions are largely free from selection (neutral) and are accumulated at a rate similar to the mutation rate. Inversely, because natural selection mainly acts on protein sequences, non-synonymous substitution accumulation is correlated with selection. If amino acid changes are neutral, non-synonymous substitutions will be fixed at the same rate as synonymous mutations and therefore ω ≈ 1. If amino acid changes are deleterious, purifying selection will reduce their fixation rate so that ω < 1. Finally, when amino acid changes offer a selective advantage, they will be fixed at a higher rate than synonymous substitutions by positive Darwinian selection, with ω > 1. We analysed the aligned nucleotide sequences using six-codon-based models described by Yang *et al*. [39]. These models explore whether, given a phylogenetic tree and a codon sequence alignment, variable selective constraints among amino acid sites (ω) are present. Model M0 assumes a single ω ratio estimated for the whole alignment. Model M1 (neutral) assumes two categories of sites in the protein: the conserved sites (w = 0) and the neutral sites (w = 1) and the proportion of sites distributed in the two classes (p0 and p1 = 1 - p0) are to be estimated from the data. Model M2 (selection) adds a third class of sites, with w2 freely estimated. Model M3 assumes 3 categories of sites with corresponding ω and proportion (p). Finally model M7 and M8 assume that ω ratios are distributed among sites according to a beta, which, depending on parameters p and q, can take different shapes in the interval (0,1). M7 does not allow for positively selected sites, whereas M8 can, by allowing an additional category of site with ω as free parameter. The ω ratio and the proportion of sites were estimated for each codon category by the maximum likelihood approach implemented in the codeml program from the paml package [40]. We used the topology presented in Figure 1C as input tree for codeml. The goodness of fit of two nested models can be compared using the likelihood ratio test: twice the log-likelihood difference between the two models (2ΔlnL = 2 × [lnL1 - lnL0]) can be compared to a χ^2 ^distribution, with the number of degrees of freedom equal to the difference in the number of free parameters between the two models. Then, to determine whether evolutionary forces acted differently on the different *Rickettsia *species, we compared the Ka/Ks ratios for the different branches in Figure 1A.

### RT-PCR assay

In an effort to determine whether *sca*1 was transcribed into mRNA, we extracted RNA from *R. conorii *using the Rneasy Mini kit (QIAGEN, Hilden, Germany). A one-step RT-PCR was performed using the Superscript one-step with Platinum *Taq *(Gibco-Brl). Within a final volume of 25 μl, we incorporated 2 μl of template RNA, 0.7 μl RT polymerase, 12.5 2-X reaction buffer, 1 μl of each of the primers s1.conoF1 and s1.conoR1 (Table 2), and 7.8 RNAse-free distilled water. On a PTC-200 thermal cycler (MJ Research), cDNA synthesis was performed by a 30-min step at 50°C, followed by a step at 95°C. Then, PCR amplification was conducted using 40 cycles made of a denaturation step at 95°C for 30 sec, an annealing step at 50°C for 30 sec, and an elongation step at 72°C for 1 min. The amplification was completed by a final 7-min elongation at 72°C. RT-PCR products were resolved on a 1% agarose gel as described above. RT-PCR products were sequenced as described above using RT-PCR primers. Distilled sterile water, processed as described above, was used as negative control. In addition, PCR performed as described above using RT-PCR primers was performed on the RNA extract to detect the presence of contaminant DNA.

## Authors' contributions

The individual parts of the work presented in the paper were conducted as follows: MN carried out the molecular genetic studies, analyzed the sequences and drafted the manuscript. GB analyzed the sequences and drafted the manuscript. PEF participated in the study design, coordinated the study, and drafted the manuscript. DR conceived the study, and helped to draft the manuscript. All authors read and approved the final manuscript.

## Figures and Tables

**Table 1:** Rickettsial strains included in this study

***Rickettsia *species**	**Strain**	***sca*1 gene**		***Sca*1 gene GenBank accession number**	***Sca*1 488-bp variable fragment GenBank accession number**
				
		**size(bp)**	**G+C%**		
*R. aeschlimannii*	MC16	5,193	34.37	AY355353	DQ306900
*R. africae*	ESF-5	5,561	33.56	AY355350	DQ306901
*R. akari*	MK (ATCC VR-148^T^)	4,614	34.09	AY355359	DQ306902
*R. australis*	Phillips	4,695	34.12	AY355360	DQ306903
*R. bellii*	369L42-1	1,782	32.1	AY355361	DQ306904
*R. canadensis*	2678	423, 2,331, and 948 ¶	32.33	AY355367	DQ306905
R. conorii *	Malish7	5,709	33.58	NC_003103	DQ306906
R. felis *	URRWXCal_2 _(ATCC VR-1525)	5,112	34.64	NC_007109	DQ306907
*R. helvetica*	C9P9	3,546	34.68	AY355363	DQ306908
*R. honei*	RB (ATCC VR-599)	5,490	33.77	AY355351	DQ306909
*R. japonica*	YM	5,928	34.02	AY355352	DQ306910
*R. massiliae*	Mtu1	5,109	34.41	AY355364	DQ306911
*R. montanensis*	M/5-6	5,910	34.40	AY355358	DQ306912
*R. parkeri*	Maculatum20	5,730	33.46	AY355354	DQ306913
R. prowazekii *	Brein1 (ATCC VR-142)	909, 1,884, and 1,044 ¶	30.15	NC_000963	DQ306914
*R. rhipicephali*	3-7-6	5,439	34.40	AY355365	DQ306915
*R. rickettsii*	R(Bitterroot) (ATCC VR-891^T^)	5,601	33.82	AY355355	DQ306916
*R. sibirica*	246 (ATCC VR-151^T^)	5,556	33.50	AY355356	DQ306917
*R. slovaca*	13-B	5,316	33.48	AY355357	DQ306918
R. typhi *	Wilmington (ATCC VR-144)	3,393	30.83	NC_006142	DQ306919

* sca1 gene sequences were retrieved from complete genome sequences in 4 species.

¶ For *R. prowazekii *and *R. canadensis*, we indicated the size of each of the three fragments of *sca*1.

**Table 2:** Oligonucleotide primers used for PCR amplification and sequencing of rickettsial species. The position of the 5' nucleotide of each primer is numbered with respect to the *sca*1 gene sequence of *R. conorii.*

**Primer name**	**Sequence in *sca*1 (5' – 3')**	**5'Position in *sca*1**
1f	ATGAATAAGTTAACAGAACA	1
2r	TGATCCTCTATAGGAACATC	335
2f	ACCCCGCAGCAGATAGGA	198
3r	TTGGTGTATTAGGTGCAGT	565
3f	AGCGTCTTCGCCTACTGT	521
4r	AAATGTCGGCTTCGGAGGA	813
4f	AGCAGTAGCGGCCCGCAA	753
5r	TTGTAACCGGAACTGTTTCCT	1198
5f	AGAAACAAAAATCAAAATAGGTGA	1031
6r	TTTCGCACTAGCAGCTACTT	1434
6f	AATCAAGCGGAGGTAATGCA	1279
7r	GGCACTAAGTACCCCTCTTCA	1667
7f	ACTCCTGTTGTTTATAATAGT	1530
8r	AACTTTCTCATTTCTGTTGCT	1851
8f	TGAAGAGGGGTACTTAGTGCC	1646
9r	AGATATTTCCGCTTGCTCA	1989
9f	AGCAACAGAAATGAGAAAGTT	1850
10r	TCTGAACGAACGCGTCGCCA	2248
10f	TGAGCAAGCGGAAATATCT	1979
11r	TCTCCGAAATACTTCCACTT	2371
11f	TGGCGACGCGTTCGTTCAGA	2228
12r	TGTTCTTTGCTTTGAGGTTTCTCCA	2616
12f	TGCGTACAGAAACTTTAGGT	2395
13r	AGGCTTATAAGCTGAGTTGCCT	2726
13f	TGGAGAAACCTCAAAGCAAAGAACA	2591
14r	TTATAAGCTTTTATATCTTCCGGTT	2894
14f	AGGCAACTCAGCTTATAAGCCT	2704
15r	ATATCCATATTAGCAGTTGGCAT	3032
15f	AACCGGAAGATATAAAAGCTTATAA	2869
16r	TATTATATATTGAAGCATCTACATCT	3220
16f	ATGCCAACTGCTAATATGGATAT	3009
17r	TTCTCATAACTAACCGACTCATA	3365
17f	AGATGTAGATGCTTCAATATATAATA	3150
18r	TTTCTTGTCAAGCTCGGCACTT	3483
18f	TATGAGTCGGTTAGTTATGAGAA	3342
19r	AGAAAATTCTGCTTCTATGGCTT	3687
19f	AAGTGCCGAGCTTGACAAGAAA	3461
20r	ATCCTCTAAGCCACTAACCGA	3816
20f	AAGCCATAGAAGCAGAATTTTCT	3664
21r	ATACCGGCTTACTCTCAGTT	3997
21f	TCGGTTAGTGGCTTAGAGGAT	3795
22r	TAGAACTTGAGCCAGACAATTCT	4201
22f	AACTGAGAGTAAGCCGGTAT	3977
23r	TTCGCGAAGTAACGAAGAAA	4350
23f	AGAATTGTCTGGCTCAAGTTCTA	4178
24r	TAATGCTAATGCACTCTCATTACTT	4521
24f	TTTCTTCGTTACTTCGCGAA	4330
25r	ATCCCCATCGGAATCGGATA	4644
25f	AAGTAATGAGAGTGCATTAGCATTA	4496
26r	TTAGACATATTCATAGATGCATCTAA	4817
s1.cono.f*	TGAGGGTATGGCGATGAGT	95
s1.cono.r*	CAGTAGGCGAAGACGCTG	502

f = forward primer; r = reverse primer; *primers used for RT-PCR.

**Table 3:** Log-likelihood values and parameter estimates for the codon-based models of nucleotide substitutions

Model	LnL	Estimates of Parameters*	Predicted positively selected sites¶
M0 (one-ratio)	-7522	ω = 0.91	None
M1 (neutral)	-7491	ω_0 _= 0 (p_0 _= 0.46) ω_1 _= 1 (p_1 _= 0.54)	Not allowed
M2 (selection)	-7461	ω_0 _= 0 (p_0 _= 0.42) ω_1 _= 1 (p_1 _= 0.48) ω_2 _= 5.96 (p_2 _= 0.01)	
M3 (discrete)	-7461	ω_0 _= 0.06 (p_0 _= 0.56) ω_1 _= 1.59 (p_1 _= 0.38) ω_2 _= 7.61 (p_2 _= 0.06)	
M7 (beta)	-7491	P = 0.024 q = 0.020	Not allowed
7M8 (beta + ω)	-7461	p = 0.024 q = 0.021 (p_0 _= 0.90) ω = 5.75 (p_1 _= 0.10)	

* Sites attributed to the highest ω category with posterior probability > 0.90.

¶p_i _is the proportion of sites included in the site category.

**Table 4:** Likelihood Ratio Test Statistics

Comparison (H_0 _vs. H_1_)	2ΔlnL	d.f.	P value
M0 vs. M1	62	1	<< 0.0001
M0 vs. M2	122	2	<< 0.0001
M0 vs. M3	122	4	<< 0.0001
M1 vs. M2	60	2	<< 0.0001
M7 vs. M8	60	2	<< 0.0001
